# A High Rate Algal Pond Hosting a Dynamic Community of RNA Viruses

**DOI:** 10.3390/v13112163

**Published:** 2021-10-26

**Authors:** Emily E. Chase, Sonia Monteil-Bouchard, Angélique Gobet, Felana H. Andrianjakarivony, Christelle Desnues, Guillaume Blanc

**Affiliations:** 1 Microbiologie Environnementale Biotechnologie, Institut Méditerranéen d’Océanologie, 163 Avenue de Luminy, 13009 Marseille, France; sonia.monteil@univ-amu.fr (S.M.-B.); harilanto.andry@mio.osupytheas.fr (F.H.A.); 2 Institut Hospitalo-Universitaire (IHU) Méditerranée Infection, 19-21 Boulevard Jean Moulin, 13005 Marseille, France; 3 MARBEC University Montpellier, CNRS, Ifremer, IRD, 34203 Sète, France; angelique.gobet@ifremer.fr

**Keywords:** microalgae, *Marnaviridae*, community diversity, community dynamics

## Abstract

Despite a surge of RNA virome sequencing in recent years, there are still many RNA viruses to uncover—as indicated by the relevance of viral dark matter to RNA virome studies (i.e., putative viruses that do not match to taxonomically identified viruses). This study explores a unique site, a high-rate algal pond (HRAP), for culturing industrially microalgae, to elucidate new RNA viruses. The importance of viral-host interactions in aquatic systems are well documented, and the ever-expanding microalgae industry is no exception. As the industry becomes a more important source of sustainable plastic manufacturing, a producer of cosmetic pigments and alternative protein sources, and a means of CO_2_ remediation in the face of climate change, studying microalgal viruses becomes a vital practice for proactive management of microalgae cultures at the industrial level. This study provides evidence of RNA microalgal viruses persisting in a CO_2_ remediation pilot project HRAP and uncovers the diversity of the RNA virosphere contained within it. Evidence shows that family *Marnaviridae* is cultured in the basin, alongside other potential microalgal infecting viruses (e.g., family *Narnaviridae*, family *Totitiviridae*, and family *Yueviridae*). Finally, we demonstrate that the RNA viral diversity of the HRAP is temporally dynamic across two successive culturing seasons.

## 1. Introduction

RNA viruses persist in a range of environments from soils [1] to seas [2,3], from the Arctic [4] to Antarctic [5], and lake waters [6,7]. The ubiquitous nature of viruses in general has been acknowledged broadly [3], however RNA viruses are historically overlooked, and it is now accepted that they may rival or succeed the amount of DNA viruses in the ocean environment based on a study of coastal waters [8]. It is unknown if this holds true spatially and temporally across other marine microbial ecosystems, but it does challenge previously held beliefs about the environmental abundance and importance of RNA viruses in comparison to DNA viruses. From around the time of this acknowledgement, to the present day there has been a considerable spike in both the number of viral RNA metagenomic studies, and the defining of new RNA viruses by the International Committee on Taxonomy of Viruses (ICTV) [9]. This growing number of metagenomic studies benefits from the increasing sequencing depths of new technologies, alongside new bioinformatic tools that are better equipped for assembling RNA viruses, including viral populations from already publicly available metagenomic datasets and studies [9]. Along with their considerable geographical range (i.e., ubiquity), RNA viruses also infect a wide range of hosts including plants [10], vertebrates [11,12], invertebrates [13,14], fungi [15], bacteria [16], archaea [17,18], and unicellular eukaryotes including microalgae [19]. The RNA viruses of unicellular eukaryotes, the so-called “protists” (when excluding fungi), are especially interesting given that many protists have remained in their mostly aquatic environment instead of transitioning to primarily terrestrial environments alongside other aforementioned hosts, consequentially, permitting the maintenance of potentially ancient viral lineages [20]. Although the prominence of viral “dark matter” (i.e., putative viral sequences that do not align to any classified virus in current databases) is widespread among viruses in general [21], the identification and study of protist viruses (including microalgal viruses) can shed important light on eukaryotic virus lineages [22] and expose previously unexplored (or understudied) viral diversity.

As an overview of diversity, RNA viruses exist in the forms of single stranded positive [(+)ssRNA] and single stranded negative sense [(−ssRNA] (e.g., Group IV, and Group V), as well as there being double stranded(dsRNA; e.g., Group III) forms [23]. Among the (+)ssRNA is the order Picornavirales [23], which includes eight families as of 2019 [23]. Historically, members of the order Picornavirales are composed of one post-translationally modified polyprotein (with the exception of family *Dicistroviridae*), similarly structured capsids and helicases, and an RNA dependent RNA polymerase (RdRp) [24], however there are many RNA viruses defined as “picorna-like”, thereby not possessing all of these exact attributes or a clear classification to date. At present, the importance of general viral interactions with microalgae in the world’s oceans is becoming clearer after RNA viral studies being overshadowed by studies of marine DNA viruses, specifically in the instance of the family *Marnaviridae* (order Picornavirales), which infect microalgae.

Among early studies of family *Marnaviridae*, the virus HaRNAV was characterized and shown to infect the toxic bloom alga *Heterosigma akashiwo* [25]. HaRNAV is among other early studies of RNA viruses infecting microalgae including RsetRNAV infecting *Rhizosolenia setigera* [26], and viruses infecting *Chaetoceros* spp. [27,28,29]. In comparison to the family *Picornaviridae* (another family in the order Picornavirales), which infect many economically important animals (e.g., cattle, birds, pigs), and humans [30], there are substantially less studies focused on family *Marnaviridae*. Microalgae are an industrially and economically important group of organisms with a variety of uses, and potential uses, relating to cosmetics [31], food and nutritional resources [32], biopharmaceuticals [33], renewable energies (i.e., biofuels) [34], wastewater treatment, and CO_2_ remediation [35]. These uses and their potential have linked microalgae with a series of “high-value” products [36]. Microalgal cultivation technologies are developed and reviewed with sustainability as an important pillar (detailed more below) [37]. Given the role of primary producers in regulation of marine nutrients [38] and geochemical cycles [3] it has been suggested that microalgae have potential as a tool for carbon sequestering [39], an important action for tackling climate change [40]. We should consider how viral studies can provide important information for future and current technological advancements in the age of sustainability (e.g., microalgal technologies). Given that elucidating new viruses and furthering our understanding of them is a major goal of the virology field, we must seek unique environments to further our studies. With this consideration we turn our primary focus to photosynthetic protists, the microalgae, which contain a potentially unexplored diversity of RNA viruses with economic importance. Viruses of microalgae, including the understudied *Marnaviridae*, have been labelled as a clear biological pollutant of microalgae culturing by cell infection [41], thereby creating a need for further studies specifically in the context of microalgae intensive culturing practices. Furthermore, viruses are important factors known to shape microbial ecosystems in general [42], so known infectants of microalgae should be studied in these culturing systems.

In consideration of the knowledge gaps within RNA viruses, especially in the *Marnaviridae* family, we carried out a study with the motive of characterizing new RNA viruses by sampling an industrial microalgal culturing basin; a high-rate algal pond (HRAP). Our goals are to (1) provide a complete view of the RNA viral diversity of the system, (2) identify new RNA viral populations, especially those that could be infecting microalgae, and (3) explore specific RNA viral population dynamics in the system to detect changes over time. We achieve goals (1) and (2) by a metagenomic study and (3) by a quantitative RT-PCR (RT-qPCR) approach.

## 2. Materials and Methods

### 2.1. Experimental Design and Sampling

Water samples for analysis were taken from a pilot microalgal polyculture in a partially open-to-air (i.e., open system) 160m^2^ HRAP (for details see [43]) basin system cultivating microalgae for CO_2_ remediation in the IFREMER (Institut Français de Recherche pour l’Exploitation de la Mer) marine station at Palavas-les-flots, France. Pure CO_2_ with a final pH of 7.5 was systematically pumped into the HRAP for the uptake of CO_2_ by these large-scale cultivations of microalgae as part of a green initiative for capturing carbon emissions expelled by industrial practices. This system featured natural colonization where seawater was pumped from the Mediterranean Sea (Plage du Prévost area) (i.e., inoculated using non-specific microalgae inoculant), and filtered through a 100 µm sand filter before entering into the HRAP. The system was restarted in the same way after each microalgal die-off (signaled by a dark green culture becoming translucent). System restarts, termed a new “run”, after microalgal die-offs occurred on 17 July 2017 and May 28, July 5, and September 20 of 2018. Raw water samples (1 L) were taken for subsequent filtration and metagenomic sequencing (i.e., viral diversity studies) on April 17, May 17, July 5, September 11, and October 23 of 2018. Additional samples (50 mL) were also taken for subsequent filtration and nucleic acid extraction for RT-qPCR viral target tracking (i.e., viral community dynamic studies) throughout the basin culturing season (approximately spring to autumn) of 2017 and 2018 (detailed further below and see Figure S1 for a timeline of sampling and HRAP runs).

### 2.2. RNA Extraction, cDNA Library Preparation, and Sequencing

Raw water samples (1 L) were clarified by centrifugation at 1500× *g* at room temperature (RT). The supernatant (800 mL) was serially filtrated at 5 µm with a Millipore Millex-SX filter, 1.2 µm with a Minisart NML Syringe Filter (Surfactant-free Cellulose Acetate) and 140 mL of the 0.2 µm eluate was concentrated into 5 mL (viral suspension) through 100 kDa TFF (Spectrum Labs PES MicroKos, Illkirch-Graffenstaden, Bas-Rin, France). Two subsamples of 1 mL were digested with a nuclease cocktail consisting in 100 µL 10× Turbo DNAase buffer (Invitrogen, Waltham, MA, USA), 40U Turbo DNase (Invitrogen, Waltham, MA, USA), 18U RNAse A, 125U benzonase, 100U exonuclease I, for one hour at 37 °C, followed by storage at −80 °C. For extraction, 3.75 mL of Trizol LS was added to 1.25 mL of the previously digested mixture, followed by homogenization and incubation for 5 min at RT. Chloroform (1 mL) was added and the mix was incubated at RT for 3 min. After centrifugation at 12,000× *g* (15 min, 4 °C) the aqueous phase was recuperated and another Trizol (3× volume)/chloroform (1.25× volume) was performed. The aqueous phase containing viral RNAs was then purified and cleaned with three columns of the Zymo Research RNA Clean & Concentrator Kit 25 using manufacturer’s instructions. The eluates (50 µL each) containing the purified RNAs were pooled (150 µL total) and 40U RNaseOUT (Invitrogen, Waltham, MA, USA) was added. RNAs were again cleaned with the Zymo Research RNA Clean & Concentrator Kit 25, 40U RNaseOUT (Invitrogen, Waltham, MA, USA) was added to the eluate (40 µL) and finally stored at −80 °C.

To achieve a library of cDNA from low concentrations of RNA we carried out a reverse transcriptase and Klenow method [44,45], with a preliminary step of denaturation at 65 °C for 5 min, with a cooling immediately afterwards. Additionally, at the end of the reverse transcriptase reaction we incubated at 94 °C for 2 min and cooled the cDNA before the Klenow reaction. We extended the Klenow incubation step by 30 min (1 h total at 37 °C). An Ampurebead (Agencourt; Beckman Coulter Life Sciences, Indianapolis, IN, USA) purification was done on the cDNA, followed by an amplification with UP primers [45]. A purification was performed by NucleoFast Ultrafiltration PCR cleanup. Concentration and quality of the sample were checked by Picogreen and Agilent DNA 7500 procedures respectively. Finally, a Nextera XT Library Prep Kit (2 × 250bp) was used for Illumina MiSeq sequencing preparation following manufacturer’s instructions and sequencing was carried out.

### 2.3. Quality Control of Reads and Contig Assembly of Metagenomes

Paired-end Illumina MiSeq reads (2 × 250 bp) were preliminarily run through fastQC [46] and results were reviewed visually to check for any overrepresented sequences, per base N content, sequencing quality scores, sequence duplications, etc. Trimming of Nextera adaptor sequences and low-quality sequences were performed by Trimmomatic v0.36 [47], followed by a secondary run of fastQC. Sequencing assemblies were produced for each metagenomic sample’s reads separately, and also as a combination of all ultravirome RNA reads (i.e., April, May, July, September, October) using the rnaviral option of SPAdes v3.15.0 [48]. Sequencing and assembly statistics on the sequencing results of each sample were calculated by QUAST [49] and are reported in Table S1. Reads were mapped back to the assemblies to estimate contig read coverage and observe any cross-sample similarities using HISAT2 [50].

### 2.4. Taxonomic Assignment of Contigs and Annotation

To study the diversity of each metagenomic sample we first performed a nucleotide-to-protein alignment [51] search using MMseqs2 [52] against the National Centre for Biotechnology Information (NCBI) non-redundant protein database (NR) [53] using an e-value cut-off of 10^−5^. Each contig with a significant match was then attributed to the same putative taxonomic clade (i.e., this method cannot define a definitive taxonomy on its own) as its best match. These results were then used to filter contigs by likely viral hits for inspection of the viral diversity of each sample.

All assembled contigs were run through EMBOSS getorf [54] to extract and translate open reading frames (ORF) of at least 30 codons. The resulting protein sequences were then searched against the NCBI NR database, and SWISS-PROT to provide a functional annotation with hits corresponding to an e-value cut-off of 10^−5^. Additionally, predicted proteins were assessed with InterProScan [55] for further functional annotations. More specifically, contigs greater than 6Kb are presented with their InterProScan [55] predicted domains annotated alongside a reference genome. The reference genomes are a curated selection of *Marnaviridae* genomes accessed through NCBI GenBank based on best matches of putative ORFs from the said contigs.

Contigs representing 99% of reads from the combined assembly of all sample reads were further investigated using the previous HISAT2 [50] reads mapping data. The number of reads mapping from each metagenomic sample back to the combined assembly (i.e., each contig) were normalized by dividing the total reads per the said sample and multiplying by 1 million. These data were used to calculate the proportion of reads mapping to specific taxonomic classifications, and also the absence of classification (i.e., a contig with no hit to a database).

### 2.5. Alpha and Beta Diversity by K-Mer Counts Produced from Metagenome Contigs

Alpha diversity indices were calculated using all contigs above 250 bp individually for each month’s metagenome sample (i.e., April, May, July, September, October) and for the combined reads assembly. Contigs were processed through MerCat [56] using k-mers of 21 bp in length. All k-mers with counts at 5 or more were assessed in the indices. The following indices are reported: Shannon’s diversity index [57], Simpson’s diversity index [58], and a richness estimator (Chao1). Bray-Curtis dissimilarity [59] (i.e., beta diversity) was also calculated between these same metagenome assemblies, using Simka [60] with a k-mer length of 21 bp.

### 2.6. Phylogenetic Reconstruction of the RNA-Dependent RNA Polymerase (RdRp) Domain

To elucidate all useful RdRp proteins from the NCBI NR database (May 2020) all protein sequences assigned as order Picornavirales were downloaded using NCBI E-utilities commands (esearch, efetch, and epost) [61], collapsed into 90% similarity by cd-hit [62], processed using HMMER tool kits (hmmsearch, hmmpress, and hmmscan) [63] against Pfam [64] to identify protein domains (e.g., conserved parts of proteins). This step was necessary for identifying RdRp within Picornavirales because they encode polyproteins (typically one in all families excluding family *Discistroviridae* possessing two), which are long proteins that undergo post translation modifications into subunits [65], consequently cut-offs between the RdRp and other proteins are difficult to pinpoint and at this scale require a consistent and objective pipeline for identification. From this processing, we identified and extracted 975 RdRp domains using a method adapted from previous research [66]. Putative proteins predicted from our RNA ultravirome MiSeq data were then searched against the 975 RdRp domain database, followed by this same protein domain finding procedure that identified 23 RdRp domains within the order Picornavirales. These 998 RdRp domain amino acid sequences were aligned using mafft v7.487 [67], gaps were adjusted with a custom TrimAl [68] (i.e., remove gaps occurring in 10% of sequences as long as at least 70% of the sequence remains), and this alignment was then used to produce a maximum likelihood tree with 1000 bootstrap replicates by IQTREE [69] using a general matrix substitution model (LG [70]) with empirical codon frequencies, and visualized using iTol v4 [71] with modifications.

### 2.7. qPCR Design for Viral Population Tracking

Predicted proteins with viral functional annotations were used to select contigs assembled from metagenomic data as individual targets for population dynamics monitoring via RT-qPCR. Target sequences were used to produce primer pairs through a Primer3 [72] Python script (Supplementary Material Note S1). PerlPrimer v1.2.3 [73] was used to check for primer-primer and self-interactions with a Gibbs free energy cut-off of −6kcal/mol to indicate whether the reaction is stable enough to reduce qPCR efficiency. All primer pairs were tested by in silico PCR, and the resulting amplicons were processed through mfold [74] to test for secondary structures significant enough to reduce qPCR efficiency (Gibbs free energy cut-off of −10 kcal/mol).

The aforementioned raw water sample aliquots (including from samples destined for metagenomic sequencing) were filtered at 5.0 µm, then 0.2 µm in preparation for extraction. Nucleic acid extractions were carried out on 200 µL of filtrate using a High Pure Viral Nucleic Acid Kit (Roche, Meylan, Isère, France) by manufacturer’s instructions except an elution volume of 40 µL was used. DNA was digested using a Turbo DNase Kit (ThermoFisher, Waltham, MA, USA), with a reaction termination using a 0.5M EDTA. Reverse transcriptase to acquire cDNA was carried out using SuperScript™ VILO™ cDNA Synthesis Kit (ThermoFisher Scientific, Waltham, MA, USA) by manufacturer’s instructions. RT-qPCR Reactions contained 0.2 µM of forward and reverse primer, 1 µL template, and 5 µL SsoAdvanced Universal SYBR 2x Green Supermix (ThermoFisher, Waltham, MA, USA), supplemented with molecular biology grade (MBG) water to 10 µL total volume. The qPCR program was run as follows: 5 min at 95 °C, then 45 cycles of 10 s at each of 95 °C, 60 °C, and 72 °C, then finalized with a melt curve to check for number of products (starting at 60 °C and going stepwise at 5 s for each 0.5 °C to 97 °C). Amplicon sizes were verified on 2% agarose gels in 1% Tris-acetate-EDTA (TAE) buffer against a 100bp BenchTop pGEM DNA Marker ladder (Promega, Charbonnières-les-Bains, Auvergne-Rhône-Alpes, France). Reactions were run in triplicate as on one 96 well plate (all samples) for each primer pair (e.g., virus target). Results were manually checked to confirm (1) amplification in the real-time visualization, (2) number of products as indicated by the melt curve, (3) cycle of quantification (Cq) values. A mean Cq value was calculated for each triplicate of sample reactions, the number of reaction cycles (45) minus the Cq value (termed the “inverse Cq”) and reported.

## 3. Results

### 3.1. Taxonomic Classification by Database “Best Hit”, Sequencing Coverage by Contig, and Domain Prediction

**Figure 1:**
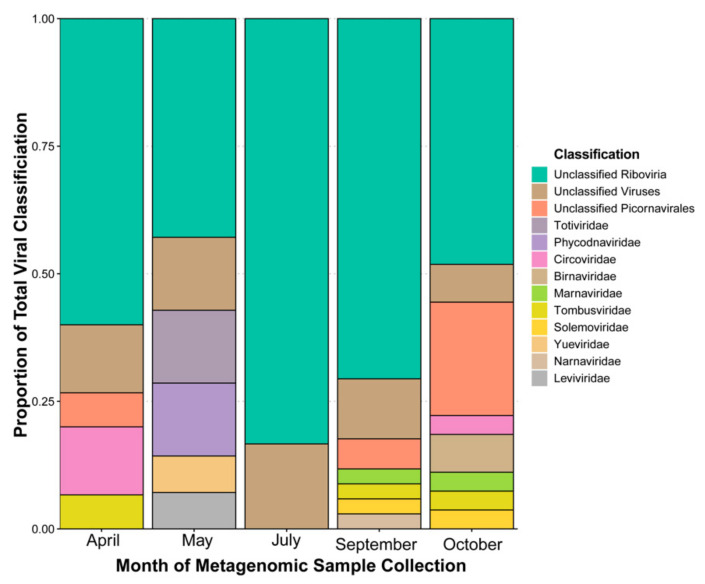
Proportion of contig viral classification (to family when possible) by each metagenomic sample month in 2018. Classifications are based on previously described database alignments by Blast. Only results of the Domain viruses are included. Total number of viral classifications by month were 15 for April, 14 for May, 18 for July, 34 for September, 27 for October.

For April, May, July, September, October, and the all-reads (i.e., all reads from each month combined and then assembled) assembly the proportions of contigs with a database hit (any classification, virus or otherwise) were 72%, 82%, 81%, 32%, 44%, and 71% respectively. Of these hits the coverage of viral assignments was 14%, 8%, 10%, 31%, 54%, and 15%. Across the 2018 monthly metagenomic samples the proportion of database viruses labelled as “unclassified” (i.e., any contigs without classification to the level of family were consider “unclassified”) accounts for over 50% of the entire sample’s hits (i.e., database hits) in all months and our combined assembly (Figure 1). Of these three categories, unclassified members of order Picornavirales appear in three months, and the *Marnaviridae* appear alongside them in two of these three months. These are potential hits of viruses infecting microalgae in our samples. Other notable hits include taxa that are known to infect fungi and oomycetes (family *Narnaviridae* [15]; family *Totiviridae* [75]), plants (family *Tombusviridae* [76]; family *Solemoviridae*), family *Birnaviridae* infecting non-mammalian vertebrates and insects, most importantly including salmon and chickens [77], a small family with species infecting freshwater isopods (family *Yueviridae* [14]), and bacteriophages (family *Leviviridae* [78]). These known hosts are not a complete picture of the potential viruses of these taxa however, and recent evidence has suggested that other RNA virus families can infect microalgae, including the families *Narnaviridae*, *Totiviridae*, and *Yueviridae* that were found in the HRAP [79]. In each of these families there are distinct hits occurring in one month exclusively. Additionally, two hits matching family *Phycodnaviridae*, a dsDNA group of nucleocytoplasmic large DNA viruses (NCLDVs [80]) for which microalgae serve as natural hosts, are detected (these include hits encoding a putative RNA polymerase large subunit and an RNA ligase with polynucleotide kinase domain). These are likely abundant enough in the basin to contribute substantial mRNA (i.e., transcriptionally active during infection [81] or encapsidated in the virion) for being detected in RNA viral targeted samples. Finally, a hit to family *Circoviridae* (a single stranded (ss) DNA virus family [82]) is detected as well, with a low similarity (28%) to a putative capsid protein.

**Figure 2:**
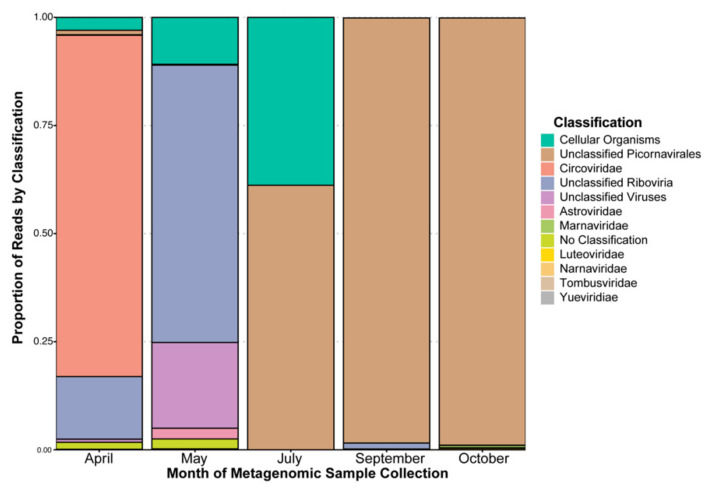
Proportion of normalised reads mapping to each classification by each metagenomic sampling month. Classifications were given to contigs assembled from all samples’ reads that accounted for at least 99% of all reads in each sample by month. These contigs included 26 viral best hits, 12 with no hit, and 496 with a cellular organism top hit. Note: due to the combining of all reads from each sample month for the assembled contigs presented in this figure additional classifications were permitted when compared to Figure 1.

When observing the proportion of reads for each classified contig (including cellular organisms and contigs without a classification (i.e., no match to a database; Figure 2)), we can attempt to infer the most relatively abundant classifications from a sequencing data perspective. In the month of April, a single contig with a *Circoviridae* hit recruits most (77%) of the reads, for May both unclassified Riboviria and “unclassified virus” hits recruit the majority of reads. For the months of July, September, and October, unclassified members of order Picornavirales dominate the read recruitments. This classification is based on only two putative full-length contigs (two viruses in theory) that recruits nearly half of the September reads each. Only one of these two contigs recruits reads in the July dataset (60%), whereas the other contig recruits most of the October reads (98%) (see Table S2).

Finally, our domain searching method applied to contigs over 6Kb uncovered several domains matching that of family *Marnaviridae* (see references genomes), including their relative order of appearance along the contig (Figure S2). Of six contigs that met the size requirement only four had adequate reference genomes available and included domains uncovered through InterProScan [55].

### 3.2. Alpha and Beta Diversity Indices, and Cross Sample Coverage

**Figure 3:**
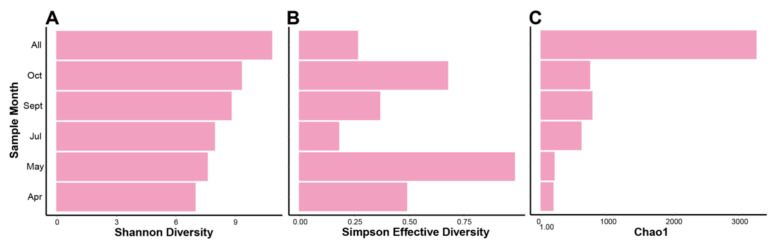
Alpha diversity indices of Illumina MiSeq metagenomic assemblies (contigs) as calculated by MerCat on k-mers of 21 bp, with a minimum count of 5 for each “k-mer species”. Indices include (**A**) Shannon’s diversity index, (**B**) Simpson’s diversity index, and finally (**C**) the Chao1 k-mer measures the number of k-mer species qualifying based on minimum count parameter. Sample months are indicated. The sample denoted “All” is an assembly of all reads from each Illumina MiSeq dataset combined.

Four intra-community diversity (i.e., alpha diversity) indices were run on k-mer based data from our assembled contigs (Figure 3). Using k-mer “species” (i.e., each unique k-mer of *n* length is treated as a single species) instead of taxonomic species, therefore freeing the data from the limitations of database dependent classification where sequencing gaps in RNA classification exist and an abundance of viruses remain as “unclassified”. A consequence of this limitation is that diversity is only quantified if the virus is “known” through a database hit, whereas using k-mers species permits an interpretation of diversity that is free from information loss when there are no hits to a database for a contig. In theory, k-mer based alpha diversity uses reoccurring k-mers as an indication of shared homology among contigs, and thusly a potential shared taxonomy.

The Shannon diversity index (Figure 3A) indicates an increase in k-mer species diversity from April to October, where unsurprisingly the combined assembly from all samples’ reads (denoted “All”) gives the highest amount of diversity. As the microalgal growing season progresses, despite microalgal die-offs occurring, the diversity in the HRAP’s viral fractions increases overtime based on k-mer species. This pattern is not reflected exactly in the Simpson diversity of k-mer species on the same samples, however it is increasing over a two-month period (April, May) and then again after a sudden decrease (July, September, October). Shannon and Simpson diversity indices are calculated in different ways (see [83]), therefore a more in-depth explanation of these results will occur in the discussion section. For the Chao1 number of estimated species (Figure 3C) we see, as expected, the highest estimate of species is in the combined reads sample. Additionally, both April and May have a lower number of estimated species, whereas July, September, and October are all relatively higher in comparison to April/May that have similar values. Based on Chao1 there is an increase in the number of k-mer species from April/May to July/September/October months.

**Figure 4:**
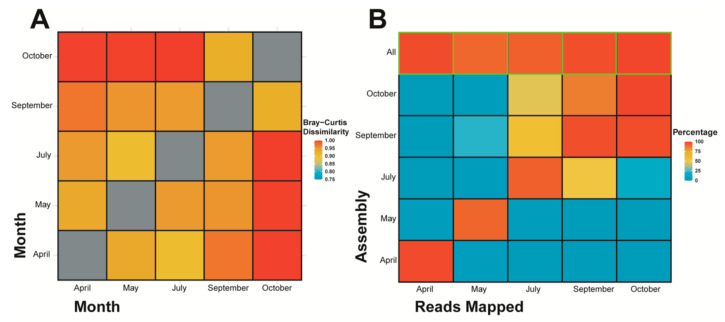
A comparison between samples done using (**A**) Bray-Curtis dissimilarity beta diversity of Illumina Miseq metagenomic assemblies (contigs) as calculated by Simka on k-mers of 21 bp, and the (**B**) percentage of reads mapped from each metagenomic sample (indicated by month of sampling) to assemblies (i.e., contigs) made from their corresponding sample month. Additionally, an assembly from all reads combined (i.e., all metagenomic samples) was produced and reads from each individual month were mapped back to the said assembled contigs (denoted by “All” and highlighted in green).

An inter-community species diversity (i.e., beta diversity) comparison using Bray-Curtis dissimilarity on k-mer species (Figure 4A) indicates that the distribution and composition of k-mer species between samples differs considerably (all values are over 0.90), however October and September are more similar when compared against other samples, additionally October individually contains more differences compared to April/May/July than September to April/May/July. July is most similar to May, whereas May and April are most similar to each other. A second interspecies comparison was done using reads mapped to contigs (Figure 4B), whereby the proportion of reads from each sample mapped to assembled contigs from each sample indicates the similarity or dissimilarity between samples. When considering all reads, and not just k-mer species (at a count of five or more) from contig assemblies as the Bray-Custis index did, October and September maintain strong similarities to one another, July has a stronger overlap with both October and September when compared with May and April, and finally May and April do not have strong similarity to one another. This last point being in contradiction to the k-mer based assessment (Figure 4A). The “all” contigs represent the assembly constructed from all samples’ reads and simply shows that a high percentage of the reads from each sample were recruited in the assembly of the combined reads contigs.

### 3.3. Phylogenetic Tree (RdRp) of Order Picornavirales

**Figure 5:**
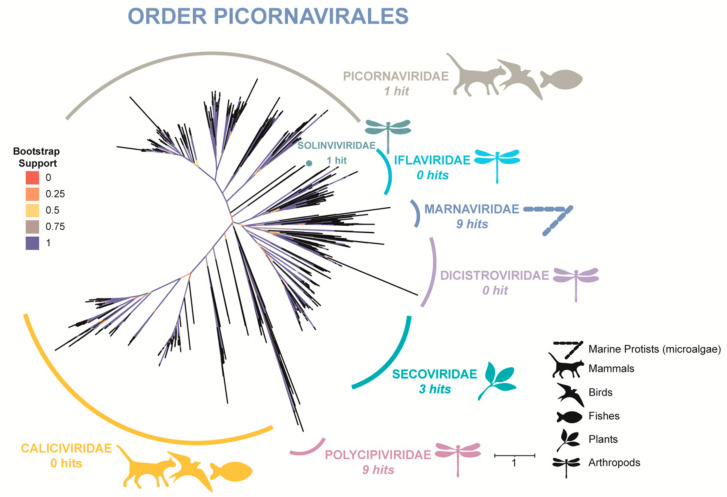
Maximum-likelihood phylogeny of RNA-dependent RNA polymerase (RdRp) of order Picornavirales based on RdRp sequences retrieved from metagenomic assembly results and the NCBI GenBank database. Bootstrap support is indicated by color, and number of RdRp hits extracted from our study are indicated alongside family classifications.

By processing nearly 39,000 sequences of published Picornavirale polyproteins and our assembled contigs we were able to form a fairly robust tree in terms of taxonomy (Figure 5). From our dataset, putative RdRp domains occur in families *Picornaviridae*, *Polycipiviridae*, *Solinviviridae*, *Secoviridae*, and *Marnaviridae*. Recovered RdRp domains from our data include nine unique sequences clustering with *Marnaviridae*, providing further evidence that this group of RNA viruses infecting microalgae not only persists in the HRAP where cultured microalgae would serve as hosts, but also as multiple *Marnaviridae* species.

### 3.4. qPCR of Potential Marnaviridae and a Rotifera sp. Virus

**Figure 6:**
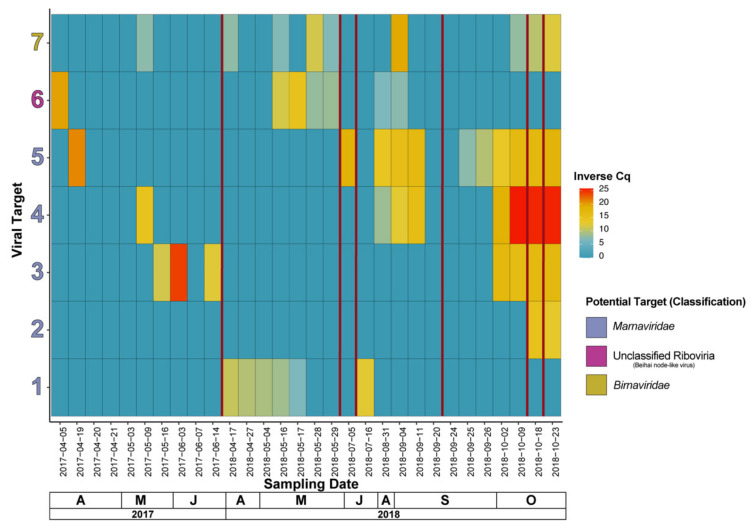
Occurrence and relative intra-species (i.e., viral population) abundance over a time series of 2017 and 2018 sampling dates, including metagenomic samples, using an RT-qPCR assay. An inverse of the quantitation cycle (Cq) is represented (45 cycles minus the Cq value), where a higher value indicates a higher relative abundance. The period between two samples where a microalgal culture die-off occurred in the basin are represented by vertical red lines (5 in total). Further information on RNA viral targets (1–7) is included in Table S3.

In total, seven RNA viruses of interest (see Table S3 for primer and putative viral target information), which were identified through taxonomic classification of contigs from the combined reads dataset, were tracked using an RT-qPCR technique. Viruses 1 to 5 in Figure 6 are potential members of *Marnaviridae*, with targets 1, 2, and 4 having a hit to a virus with an *Aurantiochytrium* sp. host, target 3 with a hit to a virus with *Cylindrotheca closterium* as a host, and target 5 with an unknown host. Additionally, target 6 has a top hit with a “Beihai noda-like virus”, and target 7 with a member of family *Birnaviridae* known to infect a member of phylum *Rotifera* and has been previously isolated before in the HRAP area in an unrelated study [84]. Two (targets 4 and 5) of the potential *Marnaviridae* virus targets share a similar pattern where infections appear to occur between the microalgae die-offs of 5 July 2018 and 20 September 2018, with persistence before, during, and after the die-offs associated with 22 October 2018. Target 2 appears only on 23 October 2018 and beyond, and finally target 1 exhibits a completely distinct pattern compared to the other potential *Marnaviridae* in which it sustains a relatively low presence during April to mid-May of 2018 and appears once thereafter. Interestingly, targets 3, 4, and 5 are all of potential *Marnaviridae*, have the most similar pattern (to a lesser extent target 3), and do not share the same host associated with their top hit. Target 6, the “Beihai noda-like virus” shows a sudden appearance and slow disappearance before the late May 2018 crash, and finally target 7, the likely *Rotifera* sp. virus comes and goes with low levels of amplification, appearing a couple of times with a relatively higher number of copies, but disappears just as suddenly. Interestingly, there are instances in 2017 dates where targets (exclusively sequenced by metagenomics from 2018 samples) are amplifying in 2017 samples also, often with a Cq implying a relatively high copy number.

Specifically, to the putative *Marnaviridae* targets (1–5) we do see variation in viral dynamics. Indeed, targets 3 to 5 are present from late September to the end of our sampling in late October, but they are not necessarily present in the same periods (with the exception of targets 4 and 5 that are quite similar). As an example, target 3 appears in October of 2018, only, but is also present in May and June of 2017, although we do not have coverage of October 2017, we can at least see that the dynamics have not repeated exactly in 2018 as they were in 2017, and therefore are not annual in our study. Target 1 has a different pattern than other putative *Marnaviridae* viruses being primarily present only in April and May of 2018.

## 4. Discussion

Our study documents the RNA viral community of an HRAP during periods of microalgae growth over two years by (1) exploring the taxonomic diversity to infer potential hosts, (2) using the RdRp gene to uncover *Marnaviridae* through a phylogenetic study, and (3) measuring dynamics of select viral targets appearing in the system over time. This study offers a unique view of an HRAP RNA virome over time that has not yet been conducted before, and can offer new insights for future microalgal cultivation, specifically in an industrial context, and in cultures which are exposed to the environment (i.e., open systems).

### 4.1. Taxonomic Classification and a Phylogenetic Study Provide Evidence of Microalgal Infecting RNA Viruses within the HRAP

Classification of viruses, in general, using metagenomics is made difficult by inconsistent coverage of taxonomic groups in databases causing what is known as “viral dark matter” where viral sequences return no hits (i.e., no alignments) to major databases hosting viral sequences [21]. In the case of RNA viruses, much is left unknown still and there are calls for more culturing and more metagenomic sequencing to advance our understanding and taxonomic classification system, despite the number of studies conducted to date [9]. This is especially important in lieu of the ICTV’s [23,85] recent acceptance of sequence-based classification of viruses, which has shown to be quite accurate through a study aimed to reproduce current viral taxonomy using gene signatures and genome organization [86]. In all but October of our metagenomic samples, the majority of hits are to non-viral classifications (e.g., “cellular organism”) despite the sample water being processed in the laboratory for targeting viral nucleic acids. Our viral dark matter ranged from 18% to 68% of the total number of contigs, with September harboring the highest number of contigs without matches to databases. When considering all non-viral hits and dark matter (e.g., “cellular organisms” are non-viral hits) also this range becomes 69% to 92%, which is not far from the ranges found in other studies (40% to 90%; [21]). Given this, we must approach our conclusions regarding the classified diversity in the HRAP with caution and understand it cannot reflect the true and complete taxonomic diversity of these samples, and therefore the HRAP itself.

With the above points in mind, we can draw some conclusions from our taxonomic analyses. Foremost, it is clear that the unclassified viruses of Riboviria, and order Picornavirales are important to this HRAP community throughout the culturing period of 2018. Of course, in regard to Riboviria, it simply means that the viruses contain either RdRp, or the RdDp (RNA dependent DNA polymerase) of retroviruses. Many of these hits are to an invertebrate focused virus study [14], which includes hosts from phyla Mollusca and Arthropoda, but remain unclassified or “picorna-like” viruses. These are unsurprising considering the water source (i.e., Mediterranean Sea) would contain Mollusca and Arthropoda species, and the open-faced nature of the HRAP in general. Among these unclassified hits is also one to a *Sclerophthora macrospora* (Oomycete) virus, which is a chimeric virus (RNA-DNA hybrid) found only in our October sample (e-value = 1.9 × 10^−16^, 57% coverage, 29% identity based on a putative RdRp sequence). With these data we observe that the HRAP is hosting a wealth of viral diversity contained in unclassified and understudied taxonomies. This includes the potential of interesting chimeric viruses that are considered a rare event and with a plausible association to microalgae as they are found in samples enriched for *Bathycoccus* [87]. Of course, within the order Picornavirales is the family *Marnaviridae*, and with hits to members of this microalgae-infecting family appearing in September and October metagenomes (2018) alongside assembled RNA contigs from the HRAP resembling the domain inclusion and domain order of known *Marnaviridae* and “marna-like” viruses (see Figure S2), we can also speculate that some of these unclassified Picornavirales could be infecting the microalgae of the HRAP as well. A recent study [79] of 570 transcriptomes from a wide diversity of marine protists revealed divergent RNA viruses considered “marna-like” due to being most closely related to *Marnaviridae* spp. yet being relatively divergent from the few species of *Marnaviridae* in genomic databases, the authors conclude that this is due to the diversity of *Marnaviridae* not yet being extensively covered and that these “marna-like” viruses are in fact *Marnaviridae* spp. This lends more evidence to the presence of RNA microalgal viruses in HRAP, which too had just been yet to be uncovered, in other words the discovery of new *Marnaviridae* in a system culturing microalga is quite plausible. Furthermore, In the same study [79] the authors used RdRp sequence and structure to identify novel microalgal RNA viruses and described them phylogenetically. Several families of RNA viruses were newly considered to be capable of infecting microalgae (based on closest relatives), and among them are the aforementioned families *Narnaviridae*, *Totiviridae* and *Yueviridae*. *Narnaviridae*-like species were found in samples of Bacillariophyta culture, and *Totiviridae*-like species were inferred to infect Bacillariophyta, Haptophyta, *Chromeraceae*, *Dinophyceae*, and Rhodophyta. Finally, a *Yueviridae*-like species was inferred to infect Bacillariophyta also. Although we cannot infer host(s) through metagenomics alone, there are many RNA viruses contained within the study samples that could be infecting microalgae. Overall, our taxonomic results reflect that of other RNA metagenomic studies and reviews in that members of order Picornavirales are common in aquatic settings [6,9,19].

By taking a normalized read mapping approach to investigate each of the 2018 metagenomic samples we can observe what viral contigs are recruiting a substantial number of reads, however, we should take into account sequencing biases produced by the process of sequencing. Although there are less studies on the subject of RNA virome sequencing biases, there is evidence of ssDNA viruses being recovered at higher proportions than dsDNA viruses [88,89] for example. Among RNA viromes there is evidence that sequencing preparation kit choice can change the profile of dominant species in wastewater samples and exclude some species that are identified in the alternative preparation kit [90]. More specific to RNA viruses that are generally A-T rich [91], this coupled with evidence that when using Nextera XT preparation kits the lower the G-C content in a region the stronger of a sequencing bias is produced [92], this is at least partially attributed to the secondary structure of RNA [93]. Given these studies, our inferences of dominant or “most important” viruses in this HRAP study must be interpreted with caution. Nonetheless, based on our read mapping and classification it is clear the viral diversity contained in the HRAP is changing across the culturing timeframe of 2018. July, September, and October featured a shared dominant viral classification (unclassified Picornavirales) that accounts for between 60% and 97% of read recruitment. Picornavirales are common in metagenomic studies of water samples, for example they were in high abundance (up to a relative abundance of 90% or more) in samples collected from groundwater in Saudi Arabia (Wadi Fatimah reservoir) [94], were up to 97% of sequence matches in a coastal water sample in Canada (English Bay, British Columbia) [95], and ultimately are considered as “widespread in the world’s oceans” [96]. Given this, it is not surprising to find that Picornavirales are dominant in read recruitment for at least three of the five metagenomes (Figure 2). With regard to the *Circoviridae* hit accounting for 77% of read recruitment in April, lake metagenomic studies have previously identified *Circoviridae* hits in RNA viromes, but concluded it was a contamination based on the presence of *Circoviridae* also in the DNA viromes [97]. An alternative explanation is that these viruses could be in an intermediary step of infection (i.e., mRNA), therefore transcriptionally active [81], given that they are ssDNA viruses and not RNA viruses [82]. Additionally, family *Astroviridae* appearing in May, are known to infect both mammals and birds [98], therefore if the natural host of this specific virus was a bird it is quite possible it could have entered into the HRAP by infected birds in the area given that it is partially open to the environment. Interestingly, contigs without hits to known databases feature low level of read recruitment, despite the seemingly high level of viral dark matter. For this case our taxonomic hits profile and our read recruitment coverage studies tell conflicting stories, however, it seems plausible that sequencing and preparation bias could contribute to the problem of understudied and unclassified viruses, alongside other factors, thereby the most “unknown” viruses are experiencing the least read recruitment in our study. Despite the aforementioned caveat regarding sequencing and preparation biases, reporting presence of the viruses identified in this study is still valid and informative.

Finally, an RdRp tree of families within the order Picornavirales were chosen for our focus because one of the seven families of Picornavirales has ample evidence of infecting microalgae, the family *Marnaviridae*. These infections have been confirmed by isolation in laboratory cultures [99], and not only through in silico analyses. With consideration to our phylogenetic tree, based on a domain searching method, we have provided further evidence of *Marnaviridae* (and thusly microalgal infecting viruses) to occur in this basin. This domain searching method was both useful in extracting RdRp domains, and in identifying putative members of *Marnaviridae*.

### 4.2. Database-Independent Approaches to Quantifying Diversity Show Changes between and among Temporal Based Samples of the HRAP

Due to the presence of viral dark matter in our study, we chose to use a k-mer based approach for measuring the alpha and beta diversity of our samples. Specifically, we chose k-mer species created from contigs and not reads to avoid library preparation and sequencing related biases, and sequencing errors contained in raw read data. Since these are k-mer species, and not taxonomic units, we cannot easily compare our results with taxonomy based alpha diversity indices of other studies but can make observations between and among our own samples. Shannon diversity is a measure of entropy/disorder in a sample that is relatively sensitive to the number of rare species present, whereas Simpson diversity has less bias in reference to the number of species sampled (e.g., k-mer species) [83]. In other words, Simpson gives less weight to rare species and more to common species, therefore more influenced by taxa evenness than Shannon. Simplified, Simpson’s index considers the probability that two species being drawn randomly from a sample will be the same, where a higher number relates to a low probability and therefore higher diversity [83]. In the context of our study, this indicates that in May where the index is high, it is relatively unlikely you will draw two of the same k-mer species, and thus the evenness among k-mers and the number of them are more consistent and higher respectively than in July for example. Given that Simpson diversity is less influenced by rare species it suggests that the number of rare or relatively uncommon k-mer species changes with time and among runs in the HRAP, but when less weight is placed on said species the diversity (Simpson) changes more drastically from month to month. Overall, there was an increase in number of different k-mer species and “disorder” (i.e., Shannon) and probability of “drawing” two of the same species of k-mers (i.e., Simpson), but when giving more weight to evenness (i.e., Simpson) we also see “spikes” in diversity in two different seasons (May; spring, October; autumn) where the increase is less gradual. These spikes, and the gradual change, do not appear tightly coordinated with die-offs in the HRAP. Overall, our alpha diversity measures indicate that the HRAP diversity is changing throughout the HRAP runs.

In the context of our beta diversity results, the diversity is different between the samples, with the dissimilarity being especially apparent between October and other months except September. There is a similar pattern when assessing similarities between samples using reads mapping, however, July also recruits over ~50% to September and October, whereas with beta diversity it’s most similar (i.e., least dissimilar) to May. Based on this study’s RT-qPCR results (discussed more below), there is evidence of viruses appearing and disappearing over time, so we cannot assume viruses appear according to season/month alone, and furthermore we cannot exclude the fact that die-offs and restarts, therefore many different runs, of the culture are occurring in the HRAP. In fact, a study focusing on a subset of dsDNA viruses (NCLDVs) showed temporal variation that did not follow a year-to-year seasonality in viral diversity [100], and another study did provide evidence of strong seasonality in a subset of marine bacteriophages (*Myoviridae*) that was linked to host seasonality [101]. Although both of these were DNA virus-based studies using metabarcoding, and ours is one of RNA and metagenomics, it is likely we cannot lump all viruses into one pattern of dynamics based on taxonomy and/or Baltimore classification and thusly can compare viral community dynamics between DNA and RNA viruses carefully to some extent. Conclusively, viral dynamics can come in many different patterns. As for the restarting of the cultures, these could easily be “resetting” the diversity with each new run, considering that the water source is from the Mediterranean Sea, and the HRAP is a small and contained body of water we likely would see different dynamics when comparing the two environments. If the HRAP takes on a different trajectory in terms of diversity and dynamics than its own water source, it would not be surprising to see a sudden shift in community diversity when the culture is restarted, and a new run occurs. This is a plausible hypothesis because a variety of factors affecting viral populations would be changed from water source to the HRAP including temperature, UV exposure, nutrient cycling, particulates, grazing by predators [102], etc. The HRAP is a constrained environment, likely selecting for different organisms in comparison to the Mediterranean Sea where the water is sourced. Although, the full study is much more complicated by this, as evident by the three restarts (i.e., four separate runs) occurring through the September and October samples resulting in October being more diverse than September. Ultimately, it is unsurprising our results based on proportion of reads mapped by classification and reads similarity between samples provide similar conclusions as they are both based on raw read data.

Results from these database independent approaches to studying diversity in metagenomics must be taken with caution. Using reads based approaches we are no doubt affected by biases introduced before and during sequencing, and with k-mer species counting we are using a relatively new method that is quite strict (k-mer species are considered different if one out of 21 nucleotides are different) and in this case it may be currently difficult to apply to metagenomes targeting taxa with high mutation rates—such are the case for RNA viruses [103]. Logically, to improve this method an approach with less strict parameters (e.g., permitting *n* number of nucleotides to be different) on contig based k-mers could improve it, and also looking at protein-based data where k-mers are created from amino acid sequences because substitutions could occur less frequently at the amino acid level than at the nucleotide level. Additionally, an obstacle of protein-based k-mer species is deciding what proteins to use and what not to use. For example, should proteins included have a specified degree of certainty in their prediction, should only taxonomically informative proteins (e.g., RdRp, capsid protein, RdDp, etc.) be included exclusively? These adjustments to the process are not mutually exclusive and conducting them in parallel might be necessary. These approaches suggested above are not available as computational tools at the time of writing, and if made available a variety of testing would need to occur for determining best practices, which is outside the scope of this study.

In general, it is not surprising that the diversity of the HRAP overall (i.e., the assembly using combined reads from all metagenomic samples) is higher than that of each month alone due to the changes witnessed in the HRAP overtime, but it does suggest that the diversity of the HRAP is better captured over time and not as a single time point. Conclusively, we insist the RNA viral diversity using metagenomics is better captured temporally in newly explored environments than as single time points.

### 4.3. Viral Dynamics (RT-qPCR Based) Showcase a Variety of Different Dynamics Patterns among Putative Marnaviridae spp., and Other Viruses of Interest

Our RT-qPCR methods in coordination with microalgae die-off dates has the potential to inform us of what viruses could be contributing or be responsible for these sudden die-offs. We used this technique to follow viruses of interest within the HRAP. We primarily focused on the *Marnaviridae* because of their host relationship with microalgae. However, the association between the appearance of these targets and the die-offs is not clear. We are also limited by our sampling, where the die-offs in May and July of 2018 lack the coverage that we gained by sampling done in September and October 2018 around their specific die-offs. We cannot completely rule out the impact of viruses on the system, however because our tracking was limited and other viruses (e.g., other RNA viruses identified in this study, and DNA viruses not studied here) could also be contributing. The effects of viral infection on microalgae may be that of several populations and other viruses in unison. Regardless, there is evidence of viruses potentially infecting microalgae appearing and disappearing temporally in the HRAP. Interestingly, three of the potential *Marnaviridae* targets appear in the 2017 samples (April, May, or June), however we do not see a gradual increase and decrease in the relative population (inferred by RT-qPCR Cq), that appears in the 2018 dynamics. These sporadic appearances could be amplification of similar strains or related viruses of the 2018 *Marnaviridae* targets, so-called “viral quasispecies”. Overall, these Picornavirale dynamics results are not unlike results obtained in a study of two Picornavirale strains among a three-lake system in America (Finger Lakes, New York), where the strains exhibited different dynamics when compared to each other and also compared across the three lakes at the same sampling times [104]. Members of order Picornavirales (and picorna-like viruses) appear to exhibit a variety of dynamics patterns, and it is unsurprising two sequential sampling years may permit different dynamics specifically given the number of runs occurring in the HRAP and the timing of them in 2017 vs. 2018.

Aside from the targets potentially infecting microalgae, the dynamics of the *Rotifera* virus are quite interesting. The relatively common *Brachionus* sp. are known predators in HRAP/microalgal culturing practices that are open faced and outdoors [105] and are documented in the Mediterranean Sea along France [106]. Given that this Rotifera virus strain has such a high certainty to be infecting Rotifera spp. (e-value = 0, 100% coverage, 99% identity), we hypothesize that *Rotifera* spp. are being reintroduced, infected, and dying off or their populations is being dramatically changed by new HRAP runs (after microalgal die-offs). We assume that the amplicon is a reflection of the relative amount of the viral copies/viruses and therefore the *Rotifera* spp. host(s) appearing in the basin, with this we might conclude a brief outbreak of *Rotifera* spp. occurred in early September 2018, and reoccurred and persisted in October of 2018. We have also assumed that this virus is a component in the regulation of the *Rotifera* spp. likely appearing in the HRAP, but we cannot exclude the importance of factors like intra-species competition and abiotic factors [100,107]. Although this virus was present before and around some of the culture die-offs, and we suppose Rotifera spp. were present alongside it, we cannot suggest if *Rotifera* spp. play a role in the die-offs in the HRAP because tracking of this microalgal grazer is not reported in this study.

## 5. Conclusions

In this study we have identified several putative RNA viruses alongside evidence to suggest that they could be infecting microalgae, specifically in the case of *Marnaviridae*, which has been uncovered through putative taxonomic classification, a phylogenetic study, and tracked in the HRAP using RT-qPCR. We cannot conclude that RNA viruses are alone responsible for the die-offs experienced in the HRAP considering we did not investigate other factors (e.g., *Rotifera* grazers mentioned above, algalcidal bacteria, other viruses not targeted in our study laboratory methods), however we have provided evidence of viruses likely infecting the microalgae being cultured in the HRAP. We have provided an overview of the taxonomic diversity of viruses contained in the HRAP, albeit limited by database completeness, alongside database independent approaches to quantifying the basin diversity. Lastly, we glimpsed into the dynamics of some viruses of interest.

In a broad sense, from our work we can conclude that future studies of RNA viruses should aim to classify environmental viruses more thoroughly alongside efforts to improve database-independent methods of studying diversity in viruses, specifically in environments not previously sampled. Advancing viral taxonomy and classification is on-going and will take time, thus improving our database independent approaches in the near future could be quite informative for viral studies similar to this. Finally, we have approached studying viral dynamics with a RT-qPCR approach, highlighting the array of viral dynamics patterns that can occur temporally and concluding that individual viruses can behave quite differently.

## Data Availability

Data are available through and hosted by the NCBI SRA portal under the BioProject I PRJNA751746, with submission ID SUB10083298.

## Supplementary Materials

The following are available online at https://www.mdpi.com/article/10.3390/v13112163/s1, Note S1: python Primer3 script, Table S1: metagenomic sample assembly statistics, Table S2: reads from each sample mapped to the metagenomic assembly (i.e., contigs) of merged sample reads, Table S3: minimum information on the putative RT-qPCR targets and primers. Figure S1: Timeline of the HRAP culturing, Figure S2: Structural analysis of putative Marnaviridae sequences.
