# Supplementary material for: A High Rate Algal Pond Hosting a Dynamic Community of RNA Viruses
Source: Viruses. 2021 Oct 26;13(11):2163. doi: 10.3390/v13112163 (PMC8619904; doi:10.3390/v13112163)
Supplement: Supplementary file 1 [file viruses-13-02163-s001.zip › Supplementary_figures.pdf]

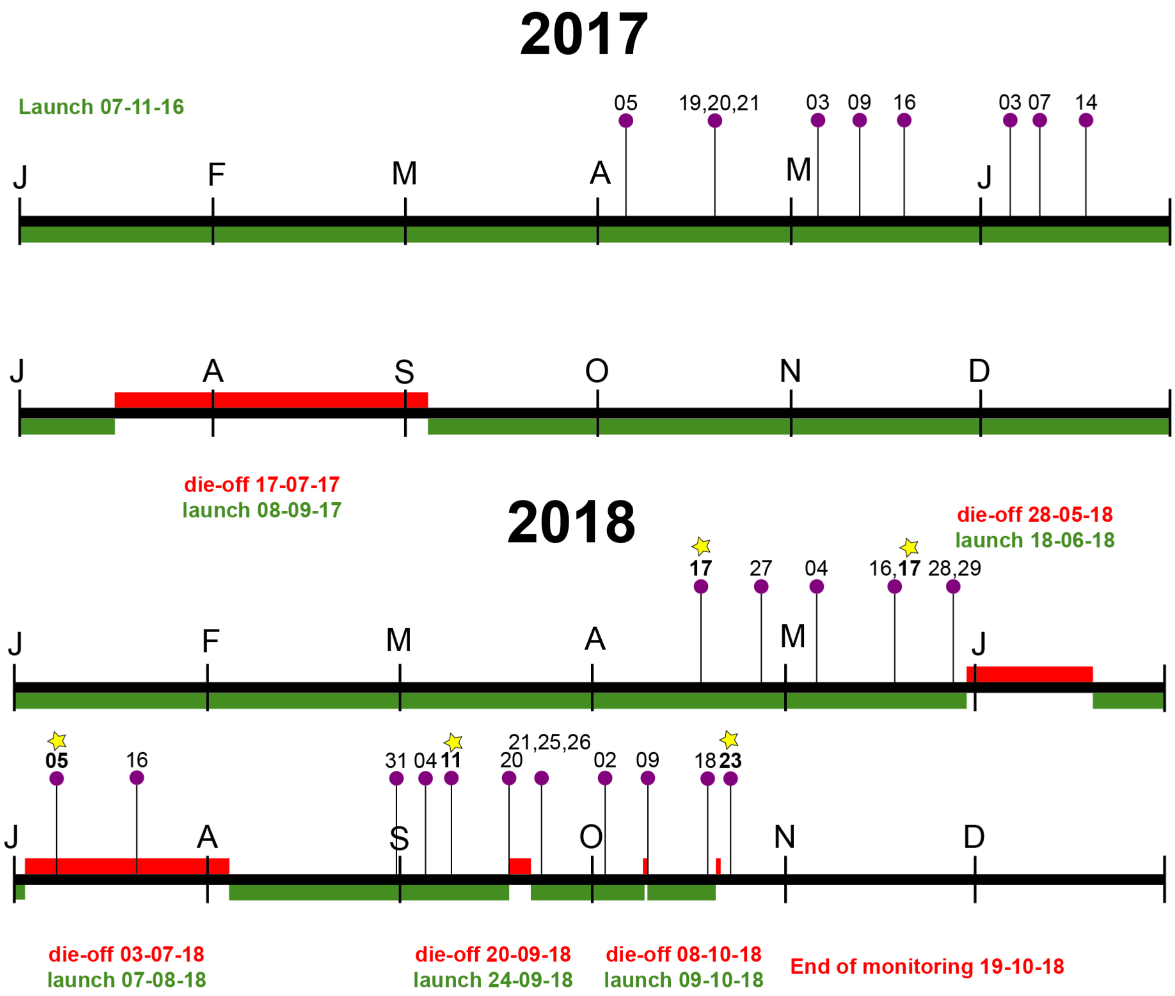

Figure S1. Relative timeline (not to exact scale) of HRAP microalgae culturing in 2017 and 2018. Culture launches and the run's span are indicated in green, where die-offs and the duration that the culture was not running are indicated in red, likewise launch and die off dates are indicated in the said colours. Purple dots indicate water sampling dates, and in cases where multiple samples were taken close together the indicator dot is shared. All dates where sample water was used for metagenomic sequencing are denoted by a yellow star. Letters indicate the sample month, in order, for each year (January to December).

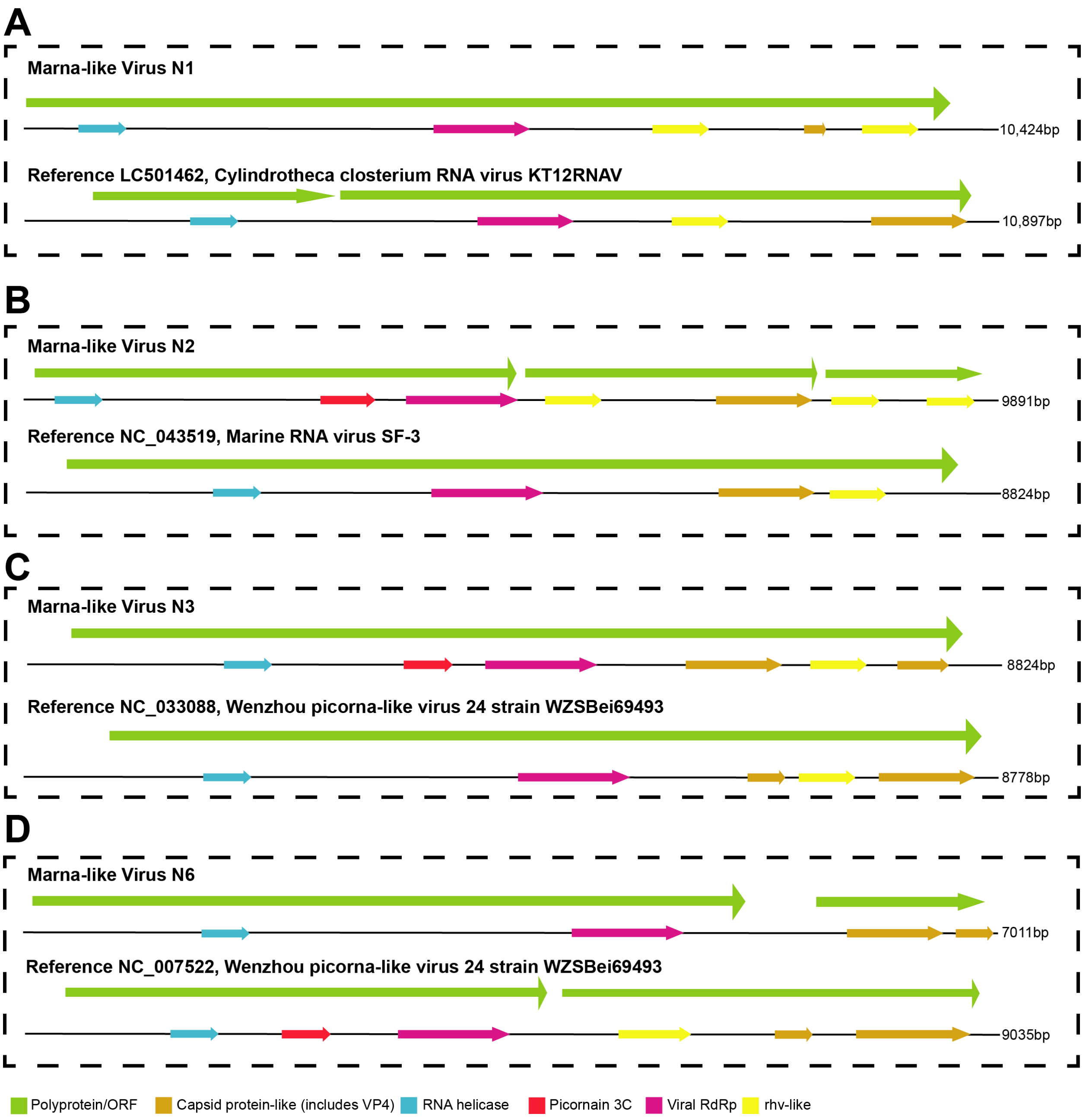

Figure S2. Structural analysis of four (**A-D**) assembled "marna-like" (i.e. putative *Marnaviridae*) viral contigs over 6Kb, with relevant GenBank matches where **A**, **B** and **D** are classified *Marnaviridae*, and **C** is a "marna-like" (listed as unclassified Riboviria on GenBank) based on their domain similarity with that of typical *Marnaviridae*. Size and placement of putative domains are relative and are for a visual representation of similarities with the reference genomes only. Putative domains are represented by a colour coded legend.
